# Oral Contraceptive Use Is Associated With Increased Tenderness to Palpation in the Masticatory System in Female Adolescents—Results of the Epidemiological LIFE Child Study

**DOI:** 10.1111/joor.70270

**Published:** 2026-07-21

**Authors:** Henriette I. U. Stasche, Gina M. Georgi, Antje Körner, Wieland Kiess, Ronald Biemann, Jens C. Türp, Christian Hirsch

**Affiliations:** ^1^ Clinic of Pediatric and Preventive Dentistry University of Leipzig Leipzig Germany; ^2^ Clinic of Orthodontics University of Leipzig Leipzig Germany; ^3^ LIFE Leipzig Research Center for Civilization Diseases University of Leipzig Leipzig Germany; ^4^ Department of Women and Child Health, Hospital for Children and Adolescents and Center for Pediatric Research (CPL) University of Leipzig Leipzig Germany; ^5^ Institute of Laboratory Medicine, Clinical Chemistry and Molecular Diagnostics Leipzig University Leipzig Germany; ^6^ Department of Oral Health & Medicine, University Center for Dental Medicine Basel UZB University of Basel Basel Switzerland

**Keywords:** female adolescence, headache, LIFE Child study, oral contraceptives, temporomandibular palpation

## Abstract

**Background:**

The utilisation of oral contraceptives (OCs) has been demonstrated to be associated with the self‐reported occurrence of headaches in female adolescents. The objective of the present study was to determine whether the ingestion of OC was also associated with increased tenderness to calibrated palpation of the masticatory system.

**Methods:**

A subsample of 111 female adolescents aged between 14 and 18 years from the LIFE Child study who were undergoing a general (with blood sample) and dental examination were included in the investigation. The presence of self‐reported headaches in the past month, as well as tenderness to calibrated palpation of the masticatory muscles and the temporomandibular joints (TMJs), was evaluated using the DC/TMD. The determination of oestrogen (E), testosterone (T), and the sexual hormone binding globulin (SHBG) was conducted through standardised laboratory analyses. In the statistical analyses, calculations were made employing logistic regression for comparisons regarding the risk experienced by female adolescents of positive tenderness to palpation and self‐reported headaches as a function of OC use. The analysis also incorporated consideration of age and body mass index (BMI).

**Results:**

The prevalence of regular OC use among female adolescents was found to be 26 out of 111 (23.4%). A subsequent analysis revealed no statistically significant disparities in age or BMI when compared to non‐users. OC use was associated with a fivefold reduction in E, a 50% reduction in T and a threefold increase in SHBG. The prevalence of self‐reported headaches and tenderness to palpation in the masticatory system was found to be significantly higher in the OC group. In the multivariable models, these relationships were confirmed, regardless of age and BMI as potential confounding factors. For self‐reported headaches, OC presented a coefficient of around six and approximately three for tenderness to palpation. Age was an additional significant factor in tenderness to palpation (OR ≈ 1.7); this correlation was not observed in cases of headaches.

**Conclusion:**

The use of OC has been demonstrated to enhance the likelihood of encountering self‐reported headaches and increased tenderness to palpation within the masticatory system in female adolescents. Consequently, the utilisation of OC in female adolescents with temporomandibular disorders warrants further consideration.

**Trial Registration:**

Ethics Committee of the University of Leipzig (Reg. No. 264‐10‐19042010) on July 10, 2010 under the clinical trial number NCT02550236. A separate positive ethical vote has been granted for the dental aspects of the trial (01/272011) (Reg. No. 354‐10‐13122010)

## Graphical abstract

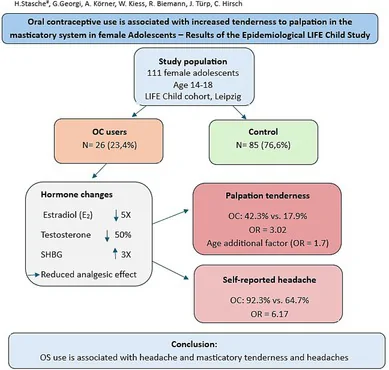

**Abbreviations**
ATC: anatomical therapeutic chemical
BMI: body mass index
BMI‐SDS: adjusted standardised body mass index
DC/TMD: diagnostic criteria for temporomandibular disorder
E2: estradiol
FAI: free androgen index
ILM: Institute of Laboratory Medicine
OC: oral contraceptives
OR: odds ratio
SHBG: sexual hormone binding globulin
T: testosterone
TMD: temporomandibular disorder
TMJ: temporomandibular joint

## 1 Introduction

The population‐based prevalence of temporomandibular disorder (TMD) symptoms is twice as high in women as it is in men [1]. Moreover, approximately 80% of TMD patients are female [2]. Among the clinically predominant signs is tenderness to palpation of the masticatory system, that is, tenderness in the masticatory muscles and/or temporomandibular joints (TMJs). The onset of pain is typically observed during the pubertal period, reaching its zenith during the reproductive years. The condition manifests most frequently among individuals between the ages of 20 and 50 [1, 2]. A multitude of factors have been advanced as contributors to this phenomenon. However, at this time, it remains challenging to provide a definitive explanation as to why women are observed to be disproportionately affected by this disorder [3].

A body of research has demonstrated that estradiol (E2) is a factor that modulates the physiopathology of orofacial pain [4]. Subsequent research has demonstrated that a polymorphism in the alpha oestrogen receptor is more prevalent in women experiencing orofacial pain [5], thereby suggesting a relationship between E levels and orofacial pain. Similar effects were also observed in males, where lower levels of free testosterone showed higher reports of tenderness to palpation in the masticatory muscles and/or TMJs [6], supporting the hypothesis that sex hormones influence pain perception.

The observation of fluctuations in masticatory muscle pain throughout the female menstrual cycle lends further substantiation to this assertion. A number of studies have demonstrated that the severity of myofascial pain escalates during the menstrual cycle, with the most pronounced prevalence and intensity occurring during menstruation [7, 8], with pain appearing to be the highest at times of lowest oestrogen [7]. This finding underscores the previously discussed relationship between oestrogen levels and musculoskeletal facial pain.

It can thus be hypothesised that there is a correlation between the use of oral contraceptives (OC) and an elevated risk of experiencing discomfort in the musculoskeletal system in the facial region. This is due to the fact that constant uptake of OC results in a persistent decrease of endogenous E2 levels. It has been shown that there is a 20% increase in the risk of suffering from TMDs for females who use OC [4] (odds ratio: 1.2 [9]). It has also been demonstrated that, especially in the instance of the initiation of an OC, there is an elevated probability of the development of a TMD, with a calculated odds ratio of 1.37 [9]. Concurrently, the probability of unsuccessful therapy for derangement in the TMJs is 2.7 times greater in females with OC usage [10].

The use of OC has been demonstrated to exert a multifaceted influence on the human organism. Beyond the immediate physical impacts, such as an elevated risk of thromboembolism [11] and worsening of migraine and tension‐type headache [12], these medications also have psychological repercussions and can lead to increased anxiety and depression [13]. It is important to note that these conditions are two well‐known bidirectional factors on TMDs as well [14].

As previously stated, research into the pubertal stage of development is always required, given its status as a pivotal stage in the development of TMDs [2, 15]. The procurement of substantial data sets pertaining to children and adolescents poses a considerable challenge within the scientific community. The acquisition of biological specimens, including the assessment of hormones through blood analysis, entails a substantial degree of compliance from patients and parents, which is not always forthcoming. Furthermore, the establishment of elevated ethical standards is a measure undertaken for the purpose of ensuring that young people are protected from unwarranted intervention; as such intervention may have the capacity to exert an adverse effect upon their development. The LIFE Child study in Leipzig, Germany, is attempting to address this issue [16]. A series of examinations were initiated in July 2011 and have continued to this day. The present analysis has involved the control of over ten thousand children and two thousand pregnant women. The overarching objective of the LIFE Child study is to enhance comprehension of the environmental, metabolic and genetic influences on child development.

The present investigation aims to further investigate the correlation between the intake of OC and tenderness to palpation in the masticatory system, as well as self‐reported headaches, with a particular focus on adolescent females utilising the data gained in the LIFE Child Study.

## 2 Materials and Methods

### 2.1 Study Participants and Setting for Examination

The LIFE Child Study is a population‐based cohort study conducted as part of the LIFE Study, a large epidemiological research project at the University of Leipzig (Germany) investigating diseases of civilisation. The present analysis comprises 111 female participants aged 14–18 years in the health cohort of the LIFE Child Study (LIFE: Leipzig Research Centre for Civilization Diseases), which has been described in detail elsewhere [16]. The study participants, comprising children and adolescents, were subjected to a thorough investigative programme. This programme encompassed dental and medical examinations, in addition to the collection of blood samples. The collection of additional information was achieved through the implementation of a questionnaire [17].

The investigation was prospectively registered with a positive vote of the Ethics Committee of the University of Leipzig (Reg. No. 264‐10‐19042010) on July 10, 2010 under the clinical trial number NCT02550236, and was conducted in accordance with the Declaration of Helsinki [18]. For the dental aspects of trial, a separate positive ethical vote (01/272011) has been granted (Reg. No. 354‐10‐13122010).

The medical and dental examinations were carried out by trained investigators in accordance with standardised protocols at the LIFE‐Child study outpatient clinic of the University of Leipzig between October 2011 and November 2014. Prior to their involvement, all subjects underwent a preliminary medical interview to ascertain their basic suitability for inclusion. Prior to undergoing the clinical examination, participants or in the case of minors, their parents, completed questionnaires regarding general health, medical history and well‐being. Consequently, a series of medical and psychological evaluations were conducted. The study incorporated female participants who demonstrated good health. The following general exclusion criteria were stipulated: a diagnosis of temporomandibular disorder (TMD), neurological or rheumatic diseases, and/or endocrine disorders. The exclusion criteria for the control group included previous use of OC or another form of hormonal contraception, such as an intrauterine device (IUD). For a comprehensive overview of the findings obtained and the biomarkers recorded in blood and urine samples, please refer to Poulain et al. [17].

It should be noted that an integral component of this procedure pertained to the dental examination. The dental examiners meticulously collated cariological, periodontal, orthodontic and function‐related findings in accordance with a predefined standard operating procedure. The investigative clinicians also performed a maxillomandibular relationship record, employing the dual‐arch impression method [19].

### 2.2 Determination of Outcome Variables

The primary outcome measure of interest was tenderness to calibrated palpation, as determined during a functional examination of the masticatory muscles and temporomandibular joints (TMJs). The examination was performed in accordance with the Diagnostic Criteria for Temporomandibular Disorders (DC/TMD) [20]. In the framework of the present study, the term ‘masticatory muscle tenderness’ was delineated as any tenderness that was identified during palpation of the temporalis (posterior, middle and anterior) and/or the masseter (origin, body and insertion) muscles. Concurrently, the term ‘TMJ tenderness’ was defined as any tenderness that was identified during palpation in or around the lateral pole. In accordance with the guidelines stipulated in the DC/TMD, palpation of the masticatory muscles and the area surrounding the lateral condylar pole was conducted with an applied force of 1 kg, while the lateral condylar pole was palpated with a force of 0.5 kg. Prior to the palpation procedure, the examiners were required to undergo a process of calibration. This training session involved the utilisation of an algometer, a device designed to measure force, with the objective of ensuring the application of force was consistent and uniform in all cases. The presence of palpation‐induced tenderness was considered a positive indication if a minimum of mild tenderness was identified at one or more sites that were palpated. In the statistical assessment, palpation findings at the masticatory muscles and/or TMJs were collated as ‘positive tenderness to palpation in the masticatory system’, irrespective of their intensity (‘mild,’ ‘moderate’ or ‘severe’) and secondary characterisation (as ‘familiar pain’ or ‘referred pain’).

The self‐reported prevalence of headaches was assessed using a dichotomous question from the DC/TMD Symptom Questionnaire. It is important to note that this measure captures patient‐reported headache occurrence and does not constitute a clinical diagnosis of a primary headache disorder. The clinical evaluation of headaches was not a component of this study; tenderness to palpation in the temporalis region cannot be equated with headache and represents a distinct finding. To this end, the patients were asked to report the occurrence of any headaches during the previous month. In the course of this investigation, the specific location, influences and first experience of the headache were assessed through anamnesis, though not through clinical evaluation.

A structured questionnaire was utilised by study assistants to meticulously document all medications taken within the 14 days prior to each participant's visit to the study centre for the assessment of OC use. Participants were requested to supply the drugs for analysis at the designated study centre. Information that was not present was collected via telephone following the visit to the study centre. Participants and their respective companions were requested to bring the medications they had been taking to the study centre. Subsequently, the drugs were subjected to scanning and documentation via a unique code or manually. An evaluation was conducted of all oral medications classified within the Anatomical Therapeutic Chemical (ATC) category designated ‚Hormonal Contraceptives for Systemic Use’ (ATC code: G03A), in addition to medications comprising the combination of cyproterone and oestrogen (ATC code: G03HB01). These medications have the capacity to be utilised for the purpose of contraception.

### 2.3 Determination of Influential Variables

In the LIFE child study, nutritional status was documented through the use of the body mass index (BMI). In order to adjust the calculated BMI for growth, the adjusted BMI was computed. The index in question served to represent the deviation of individual BMI from the age‐ and sex‐adjusted norm BMI. The analysis employed the adjusted BMI‐SDS as a dichotomous variable, categorised as below, equal to or above the 50th percentile [21].

The analysis of hormone levels was achieved through the collection of venous blood samples in the morning (7:30 AM to 10:00 AM) under fasting conditions to achieve the highest possible standardisation. Hormone analyses were conducted at the Institute of Laboratory Medicine, Clinical Chemistry and Molecular Diagnostics, University of Leipzig. The quantification of E2 and testosterone (T) was performed using liquid chromatography–tandem mass spectrometry (LC–MS/MS); a detailed description of the analytical method is provided by Gaudl et al. [22]. The measurement of sex hormone–binding globulin (SHBG) was performed using an electrochemiluminescence immunoassay on a Roche cobas e602 analyser (Roche Diagnostics GmbH, Mannheim, Germany).

A subset of the samples was cryopreserved in the LIFE Biobank. The estimation of free E2 was derived from the relationship between total E2 and SHBG, utilising the free androgen index (FAI).

### 2.4 Statistical Analyses

A comparative analysis was conducted among female participants who used an OC and those who did not, with the objective of assessing differences in age, BMI, BMI‐SDS and hormone levels (Oestrogen, SHBG, testosterone, FAI) and headache prevalence and palpation pain. The statistical analysis employed a *t*‐test for continuous variables and the chi‐square test for categorical variables (Table 2).

Multivariable logistic regression analyses with a 95% confidence interval were performed to determine the odds (as odds ratios [ORs]) of self‐reported headaches as a function of OC use. The impact of age and BMI‐SDS was controlled for through appropriate adjustments (Table 3). The same methodology was employed for the tenderness to palpation variable (Table 4).

## 3 Results

The present study encompasses a total of 111 female adolescents from the LIFE Child cohort, with ages ranging from 14 to 18. Of those, 26 (23.4%) reported regular use of OC (Table 1). A comparison of age and BMI between the two groups revealed no significant differences (Table 2).

**TABLE 1:** Distribution of female adolescents using oral contraceptives in relation to age.

Variable	Age*				Total
	14	15	16	17+	
OC yes	12.8% (*N* = 5)	26.7% (*N* = 8)	37.5% (*N* = 12)	10.0% (*N* = 1)	23.4% (*N* = 26)
OC no	87.2% (*N* = 34)	73.3% (*N* = 22)	62.5% (*N* = 20)	90.0% (*N* = 9)	76.6% (*N* = 85)
Total	35.2% (*N* = 39)	27.0% (*N* = 30)	28.8% (*N* = 32)	9.0% (*N* = 10)	100% (*N* = 111)

* Chi square test *p* = 0.092.

**TABLE 2:** Comparison of the female subjects (*N* = 111) with or without oral contraceptive (OC) use regarding influential and outcome variables.

Variables	Total	OC yes	OC no	*p*
	(*N* = 111; 100%)	(*N* = 26; 23.4%)	(*N* = 85; 76.6%)	
Age (mean ± SD)^a^	15.6 ± 1.1	15.9 ± 0.9	15.5 ± 1.1	n.s.
BMI (mean ± SD) [kg/m^2^]^a^	22.3 ± 5.5	21.2 ± 2.4	22.6 ± 6.1	n.s.
BMI SDS ≥ 0% (*N*)^b^	59.0 (161)	53.2 (33)	60.7 (128)	n.s.
Estradiol (mean ± SD) [pmol/L]	**255.0 ± 296.9**	**66.6 ± 94.9**	**312.6 ± 313.6**	**< 0.001**
SHBG (mean ± SD) [nmol/L]^a^	**100.9 ± 93.9**	**218.9 ± 98.1**	**64.9 ± 55.6**	**< 0.001**
Testosterone (mean ± SD) [nmol/L]^a^	**0.96 ± 0.55**	**0.56 ± 0.45**	**1.05 ± 0.53**	**0.0002**
Free estradiol (mean ± SD) [pmol/L]^a^	**5.49 ± 7.02**	**0.48 ± 0.76**	**7.01 ± 7.39**	**< 0.001**
Headache % (*N*)^b^	**71.2 (79)**	**92.3 (24)**	**64.7 (55)**	**0.007**
Palpation pain % (*N*)^b^	**23.6 (26)**	**42.3 (11)**	**17.9 (15)**	**0.01**

*Note:* Statistically significant values are marked in bold.

^a^ *t*‐test.

^b^ Chi‐square‐test.

However, a notable disparity in hormone levels was observed, with E2 and free E2 concentrations diminished by approximately five to 14‐fold compared with patients not using OC. Testosterone, which is of lesser relevance in females, demonstrated levels up to 50% lower, while SHBG concentrations exhibited an increase of up to threefold.

The prevalence of both outcomes examined as target variables, namely self‐reported headaches and tenderness to palpation in the TMJs and/or masticatory muscles, was significantly more common in girls who were using OC. In the multivariable models (logistic regression), these relationships were confirmed. The OC use demonstrates a positive coefficient when analysed independently, as well as when adjusted for age and age in conjunction with BMI (Table 3). The prevalence of self‐reported headaches was found to be up to six times higher.

**TABLE 3:** Multivariable analyses for the probability of headache reports among female subjects with and without contraceptive use in regard to influential variables.

Variables	Coefficient	95% CI	*p*
	Without adjusting		
OC	**6.54**	**1.44–29.6**	**0.015**
	Adjusted for age		
OC	**6.18**	**1.39–28.1**	**0.018**
Age (per year)	1.24	0.82–1.86	n.s.
	Adjusted for age, and BMI		
OC	**6.17**	**1.35–28.2**	**0.019**
Age (per year)	1.19	0.79–1.80	n.s.
BMI‐SDS (≥ 0 vs. < 0)	0.81	0.58–1.12	n.s.

*Note:* Statistically significant values are marked in bold.

Analogous outcomes were noted in the tenderness to palpation assessment. The coefficient of determination of the OC use is 3.37 when analysed independently, indicating a positive relationship (Table 4). The age of the subjects was an additional significant factor in tenderness to palpation; that is to say, older girls reported higher levels of tenderness to palpation (OR approximately 1.7). However, this correlation was not observed in the context of self‐reported headaches. In contrast, the BMI appears to be non‐significant.

**TABLE 4:** Multivariable analyses of the risk of positive pain reports on palpation of the female subjects with and without contraceptive use in relation to influential variables.

Variables	Coefficient	95% CI	*p*
	Without adjusting		
OC	**3.37**	**1.29–8.79**	**0.013**
	Adjusted for age		
OC	**3.14**	**1.17–8.43**	**0.023**
Age (per year)	**1.72**	**1.12–2.65**	**0.013**
	Adjusted for age, and BMI		
OC	**3.02**	**1.12–8.12**	**0.029**
Age (per year)	**1.71**	**1.10–2.64**	**0.016**
BMI‐SDS (≥ 0 vs. < 0)	0.83	0.54–1.27	n.s.

*Note:* Statistically significant values are marked in bold.

## 4 Discussion

The present findings suggest a correlation between the use of OC by female adolescents and an increased self‐reported prevalence of headaches as well as heightened tenderness to calibrated palpation in the masticatory system. This phenomenon has not yet been documented in this particular age group. The underlying mechanism appears to involve a decrease in endogenous E2 and T levels induced by hormonal OC. This effect was identified in a randomly selected sample of subjects from a representative population survey (LIFE Child), which largely ruled out the possibility of selection bias. The results of this population study clearly demonstrate the existence of hormonal effects on tenderness to palpation in the masticatory system, which underscores the necessity of meticulously recording the use of OC in the medical histories of the patients. This finding could also be relevant for patients suffering from TMDs.

The data presented indicated that just under a quarter of female adolescents were using OC for the purposes of contraception or therapy of other conditions, such as acne or menstrual cycle irregularities. This proportion is marginally lower than that observed in the investigation conducted by Lewandowski et al. [23]. However, it is crucial to acknowledge that our investigation included individuals aged 14 years, and as the intake increases with each year of teenagehood [24], this discrepancy can be ascribed to the varying age groups considered in each study.

The control group exhibited approximately fivefold higher E and nearly double the T values, while concurrently demonstrating only one‐third of the SHBG value. This phenomenon can be elucidated by reference to the constant synthetic oestrogen intake of the OC group. The intake has been demonstrated to induce a down‐regulation of endogenous hormone production of E and T, while concurrently stimulating SHBG production in the liver [25].

The prevalence of tenderness to palpation in the control group of 17.9% aligns with the findings reported in the extant literature [3, 26]. A comparable phenomenon was observed for self‐reported headaches, which were reported by 64.7% of female control patients [27, 28]. This finding serves to substantiate the reliability of the data presented. It must be emphasised that the evaluation of headache in this study was exclusively via self‐report, employing the DC/TMD Symptom Questionnaire. No clinical diagnosis of a primary headache disorder, such as migraine or tension‐type headache, was conducted. Tenderness to palpation in the temporalis region is a distinct finding and cannot be equated with headache. The relationship between self‐reported headaches and OC use is a multifaceted one, with a complex interplay of factors contributing to its development. The utilisation of OCs as a therapeutic modality for the management of migraine has been documented in the extant literature [29]. However, it has also been observed that OCs can, in some cases, result in the exacerbation or initiation of headaches and migraines [12]. A substantial surge in the prevalence of migraine was observed during the nascent phases of OC initiation [29, 30]. It can be hypothesised that, given the young age of our patients, they are likely to be in the first stage of OC intake. This finding provides a plausible explanation for the high prevalence of headaches (92.3%) reported by female patients using OC, as indicated in the present study.

In the context of the multivariable analysis, a significant coefficient of up to 6.54 was observed for self‐reported headaches in relation to OC use, which is notably higher than the OR of 1.34 reported in the investigation by Bianchin et al. [28]. In addition, an OR for tenderness to palpation in the masticatory system with a maximum score of 3.4 was presented. The present study has added the demonstration that the use of OC does regulate the prevalence of tenderness to palpation in the masticatory system in adolescents, a finding that has previously been established only in adults [9]. Furthermore, the present investigation corroborated the finding of Song et al. [15] that age is a negative factor for tenderness to palpation in the masticatory system. However, the occurrence of self‐reported headaches was not influenced by age, thereby allowing us to hypothesise that a distinct pathology must be present for both conditions. The concept that oestrogen exerts a favourable effect on pain modulation is well established. A robust correlation has been demonstrated between oestrogen and visceral nociception sensitivity [31]. Moreover, it is broadly acknowledged that oestrogen plays a crucial role in sensory processing [32]. The manifestation of chronic pain within the musculoskeletal system has been linked to the absence or insufficiency of oestrogen [33]. This condition warrants consideration within the context of the pathological development of TMDs and headaches. The precise mechanisms through which E2 modulates tenderness to palpation are increasingly understood: oestrogen receptors (ERα and ERβ) are expressed in the trigeminal ganglion, masticatory muscles and TMJ tissues, where E2 modulates nociceptor sensitivity and the release of pro‐inflammatory mediators. Therefore, OC‐induced suppression of endogenous E2 may result in a reduction of the analgesic effect of oestrogen at both the central and peripheral levels. Consequently, this may lead to a decrease in the threshold for tenderness in the masticatory system. Furthermore, the presence of OC has been demonstrated to suppress free testosterone, a finding that has been shown to be independently associated with reduced tenderness to palpation in males [6]. This observation suggests the potential involvement of convergent hormonal pathways. The present research thus provides further evidence that female sex hormones are involved in the modulation of tenderness to palpation, contributing to the complex interplay between hormonal regulation and pain perception [4]. Our findings demonstrate that lower E2 levels, specifically those that are induced by OC, are associated with increased tenderness to palpation in the masticatory system.

A further point warranting clarification concerns the nature of the outcome measure employed in this study. Tenderness to calibrated palpation, as determined by the DC/TMD examination protocol, is not a peripheral or ancillary finding in the context of TMD diagnosis. Rather, it is the defining clinical criterion. According to the DC/TMD, the gold standard diagnostic framework for TMDs, a positive diagnosis of myalgia and TMJ arthralgia, the two most prevalent pain‐related TMD subtypes, requires the confirmation of pain elicited or modified by examiner palpation as the central clinical sign [20]. In the absence of a positive palpation finding, neither myalgia nor arthralgia can be diagnosed under the DC/TMD protocol. Consequently, the measurement of tenderness to calibrated palpation in the masticatory muscles and TMJs, as performed in the present investigation using standardised DC/TMD procedures, represents the operationalised diagnostic marker for pain‐related TMDs in epidemiological research. This methodology is entirely consistent with the approach employed across a substantial body of population‐based TMD studies. The present findings should therefore be interpreted not as a measure of a lesser or indirect outcome, but as a direct assessment of the hallmark clinical sign underpinning the diagnosis of pain‐related TMDs.

It must be acknowledged that the present investigation is not without limitations. A psychosocial characterisation of the participants according to Axis II was not performed in this analysis. Adolescent subjects who exhibit tenderness to palpation demonstrate altered psychosocial characteristics [34]. However, the present study was conducted with the objective of identifying biological and hormonal causes for this pain condition. Psychosocial characteristics (e.g., the presence of a negative mood or depression) are not considered to be confounders, as it is assumed that hormone status influences the psyche and not vice versa. In this particular context, psychosocial characteristics represent a secondary pathway and they have been excluded from the statistical analysis. As demonstrated by Akinkugbe et al. [35, figure 1c], sex hormones have been shown to directly influence temporomandibular pain. In contrast, psychosocial factors such as hypervigilance, psychological stress, catastrophising and facial injuries do not appear to act as direct confounders in the relationship between sex hormones and TMD‐related pain.

A further limitation is that the examination results of the DC/TMD have not been subject to specification with regard to location or severity of palpation‐induced tenderness. Conversely, a dichotomous analysis was employed. Moreover, given the inability to exclude the potential for concomitant pathological processes, such as bruxism or TMJ arthralgia, the diagnostic certainty of positive tenderness to palpation findings remains uncertain. Nevertheless, this is not regarded as a substantial deficiency, considering that the central question of the present article was whether there is an association between OC usage and tenderness to palpation in the masticatory system in female adolescents. Furthermore, patients who had previously sought therapy for temporomandibular pain, that is, pain in the masticatory muscles and/or TMJs, were excluded from the study.

Finally, it is imperative to acknowledge the potential influence of fluctuations in female hormones during the menstrual cycle on the blood hormone measurements obtained on the day of the examination. The analysis did not incorporate the patients' current cycle phase. However, the statistically significant difference between the groups (*p* < 0.001) far exceeds the natural fluctuation of sex hormones and SHBG [36], thereby demonstrating the profound influence that OCs exert on the hormone status of female adolescents and thereby eliminating any concerns that the missing information should affect the results of this study. The inquiries are meticulously designed to evaluate the self‐reported prevalence of headaches, soliciting information about pain experienced over the preceding 30 days. This approach encompasses the duration of a complete menstrual cycle, thereby providing a comprehensive assessment of the subject's headache history.

## 5 Conclusion

The use of oral contraceptives has been associated with an increased risk of self‐reported headaches and heightened tenderness to palpation in the masticatory system in female adolescents. The presence of elevated estradiol (E2) levels has been demonstrated to be associated with a reduced risk of experiencing headaches and tenderness to palpation in the masticatory system.

## Author Contributions

**Antje Körner:** funding acquisition, data curation, resources, project administration. **Henriette I. U. Stasche:** writing – original draft, writing – review and editing, visualization. **Wieland Kiess:** funding acquisition, data curation, resources, project administration. **Jens C. Türp:** writing – original draft, writing – review and editing. **Christian Hirsch:** conceptualization, formal analysis, methodology, investigation, supervision. **Ronald Biemann:** data curation. **Gina M. Georgi:** validation, visualization.

## Funding

This research is funded by the Leipzig Research Centre for Civilisation Diseases (LIFE) at the University of Leipzig and the German Research Foundation (Grant DFG: HI 707/3‐1). LIFE receives financial support from the European Union, the European Regional Development Fund and the Free State of Saxony as part of the Excellence Initiative.

## Conflicts of Interest

The authors declare no conflicts of interest.

## Data Availability

The data that support the findings of this study are available from the corresponding author upon reasonable request.
